# Deep learning-based reconstruction on cardiac CT yields distinct radiomic features compared to iterative and filtered back projection reconstructions

**DOI:** 10.1038/s41598-022-19546-1

**Published:** 2022-09-07

**Authors:** Sei Hyun Chun, Young Joo Suh, Kyunghwa Han, Yonghan Kwon, Aaron Youngjae Kim, Byoung Wook Choi

**Affiliations:** 1 Department of Radiology, Severance Hospital, Research Institute of Radiological Science, Center for Clinical Imaging Data Science, Yonsei University College of Medicine, 50-1 Yonsei-ro, Seodaemun-gu, Seoul, 03722 Korea; 2 Department of Biostatistics and Computing, Yonsei University Graduate School, Seoul, Korea; 3 Weill Cornell Medicine-Qatar, Doha, Qatar

**Keywords:** Cardiovascular diseases, Biomarkers, Cardiology, Machine learning

## Abstract

We aimed to determine the effects of deep learning-based reconstruction (DLR) on radiomic features obtained from cardiac computed tomography (CT) by comparing with iterative reconstruction (IR), and filtered back projection (FBP). A total of 284 consecutive patients with 285 cardiac CT scans that were reconstructed with DLR, IR, and FBP, were retrospectively enrolled. Radiomic features were extracted from the left ventricular (LV) myocardium, and from the periprosthetic mass if patients had cardiac valve replacement. Radiomic features of LV myocardium from each reconstruction were compared using a fitting linear mixed model. Radiomics models were developed to diagnose periprosthetic abnormality, and the performance was evaluated using the area under the receiver characteristics curve (AUC). Most radiomic features of LV myocardium (73 of 88) were significantly different in pairwise comparisons between all three reconstruction methods (*P* < 0.05). The radiomics model on IR exhibited the best diagnostic performance (AUC 0.948, 95% CI 0.880–1), relative to DLR (AUC 0.873, 95% CI 0.735–1) and FBP (AUC 0.875, 95% CI 0.731–1), but these differences did not reach significance (*P* > 0.05). In conclusion, applying DLR to cardiac CT scans yields radiomic features distinct from those obtained with IR and FBP, implying that feature robustness is not guaranteed when applying DLR.

## Introduction

Cardiac computed tomography (CT) is widely used to diagnose obstructive coronary artery disease and evaluate intra-cardiac structures^1–4^. Cardiac CT allows visual analysis and quantitative parameters about cardiovascular structures such as coronary plaques, peri-coronary fat tissue, myocardium, and intra-cardiac mass^5–9^. Radiomics is a high throughput approach that extracts of high-dimensional quantitative information from digital medical images. Data obtained from radiomics provides more comprehensive and quantitative analysis than visual assessment or conventional quantitative parameters. Radiomic features derived from cardiac CT images contribute additional diagnostic or prognostic value to conventional quantitative CT parameters^10–13^.

However, concerns have been raised about the reproducibility or robustness of radiomic features due to effects such as observers who perform segmentation, image acquisition, and reconstruction parameters. For example, iterative reconstruction (IR) can potentially affect radiomic features because it reduces image noise and changes the appearance and texture of CT images^14–16^. Recently, deep learning-based reconstruction (DLR) methods have been developed that achieve more dose reduction and faster reconstruction than IR while preserving the image quality and texture obtained with filtered back projection (FBP)^17^. DLR is expected to overcome the limitations of unnatural image appearance and texture that occur with IR. DLR improves image quality compared with FBP or IR^18–20^ and facilitates dose reduction while maintaining the image quality and diagnostic performance of CT scans^21^. The effect of other deep learning-based technology, for example, deep learning-based conversion of reconstruction kernel, on the reproducibility of radiomic features has been studied before^22,23^. However, the effects of DLR on radiomic features have not yet been compared to other reconstruction methods.

The purpose of this study was to evaluate and compare the effects of DLR with IR and FBP cardiac CT reconstruction algorithm on the radiomic features of the myocardium and in diagnosing postoperative periprosthetic pathology.

## Results

### Study population

**Figure 1:**
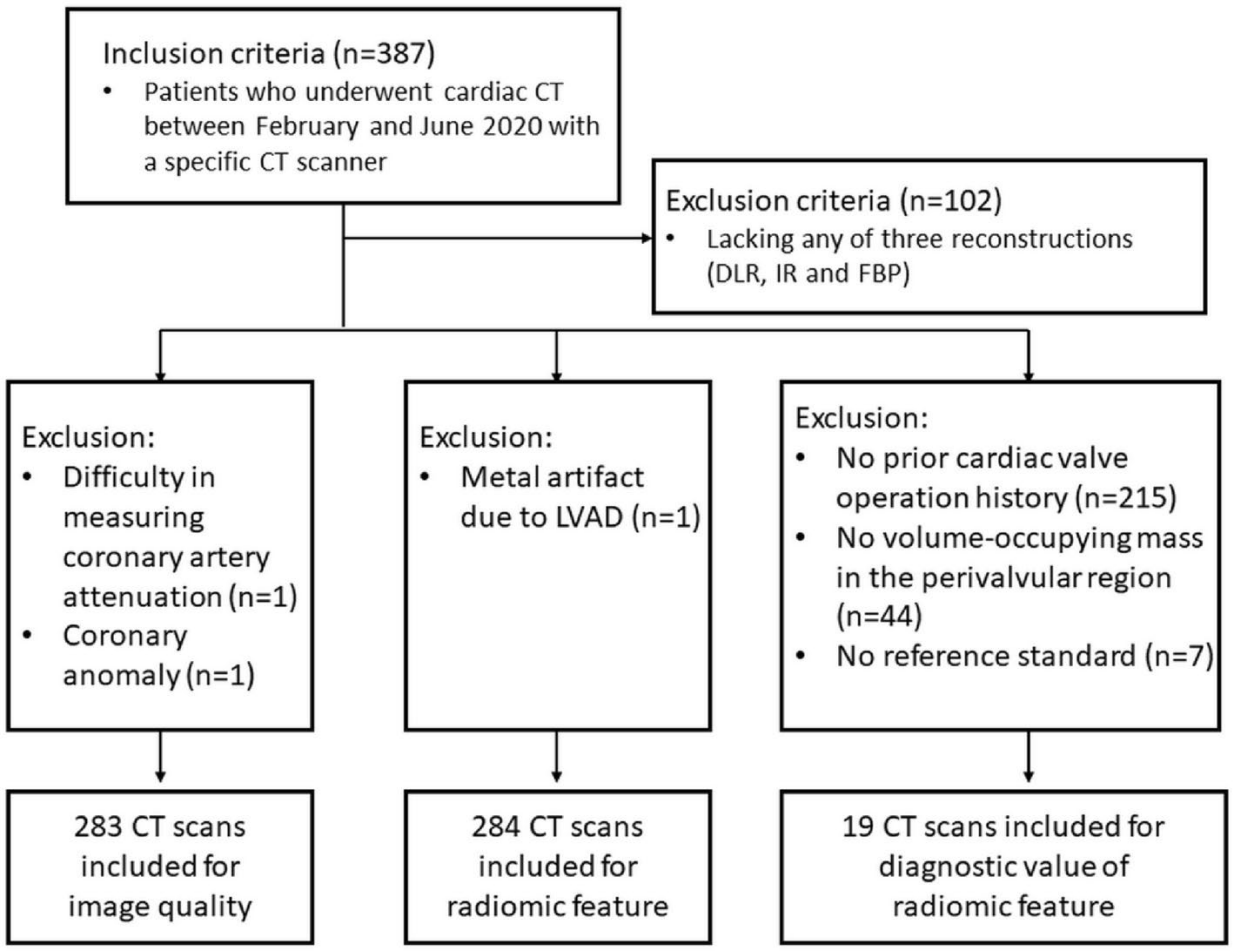
Flow chart for patient enrollment. LVAD, left ventricular assist device; DLR, learning-based reconstruction; IR, iterative reconstruction; FBP, filtered back projection.

Two CT scans were excluded from the comparison of image quality because measuring coronary artery attenuation values was difficult due to a severe artifact on the coronary artery (n = 1) and a congenital coronary anomaly (n = 1) (Fig. 1). A total of 283 CT scans from 282 patients (133 men and 149 women; mean age, 65.9 ± 12.4 years) were included in the CT image quality analysis. We excluded one CT scan from comparing myocardium radiomic features because of a metal artifact from an LV assist device. A total of 284 CT scans from 283 patients (133 men and 150 women; mean age, 66.0 ± 12.4 years) were included to compare myocardium radiomic features. We identified a subgroup of 68 patients who had undergone prior cardiac valve operation to compare the value of radiomic features in diagnosing periprosthetic masses. Among this subgroup, 19 CT scans from 18 patients (10 men and 8 women; mean age, 75.8 ± 4.2 years; Supplemental Table S1) were included because their CT scans showed volume-occupying masses in the perivalvular region. In total, 29 ROI of periprosthetic masses were segmented, and the final diagnoses of the periprosthetic masses were normal (n = 2; 4 ROI), degeneration (n = 3; 3 ROI), thrombus (n = 10; 18 ROI), and pannus (n = 4; 4 ROI). Mean dose length product of cardiac CT was 287.1 ± 91.3 mGy ∙ cm.

### Comparison of CT image quality parameters between reconstruction methods

The mean CT attenuation values of coronary arteries, image noise, SNR, and CNR are shown in Table 1. All image quality parameters differed significantly between reconstruction groups (*P* < 0.001). DLR, followed by IR and FBP, showed the lowest noise (32.4 HU vs. 60.0 HU vs. 81.6 HU), the highest SNR (20.4 vs. 10.6 vs. 8.0), and the highest CNR (22.4 vs. 11.9 vs. 8.7).

**Table 1:** CT image quality parameters for each reconstruction method.

Image quality	DLR	IR	FBP	*P *value
CTNo (HU)	623.882 (601.032, 646.731)	610.857 (587.969, 633.746)	612.344 (589.516, 635.171)	< 0.001
Noise (HU)	32.391 (29.673, 35.11)	59.961 (57.244, 62.679)	81.628 (78.903, 84.353)	< 0.001
SNR	20.36 (19.929, 20.792)	10.593 (10.162, 11.024)	7.977 (7.545, 8.408)	< 0.001
CNR	22.384 (21.742, 23.025)	11.899 (11.206, 12.592)	8.737 (8.053, 9.421)	< 0.001

Data are presented as mean values with the 95% confidence interval in parentheses.

DLR, deep learning-based reconstruction; IR, iterative reconstruction; FBP, filtered back projection; CTNo, mean CT attenuation values of left main and right coronary arteries; SNR, signal-to-noise ratio; CNR, contrast-to-noise ratio.

### Comparison of myocardial radiomic features between reconstruction methods

**Figure 2:**
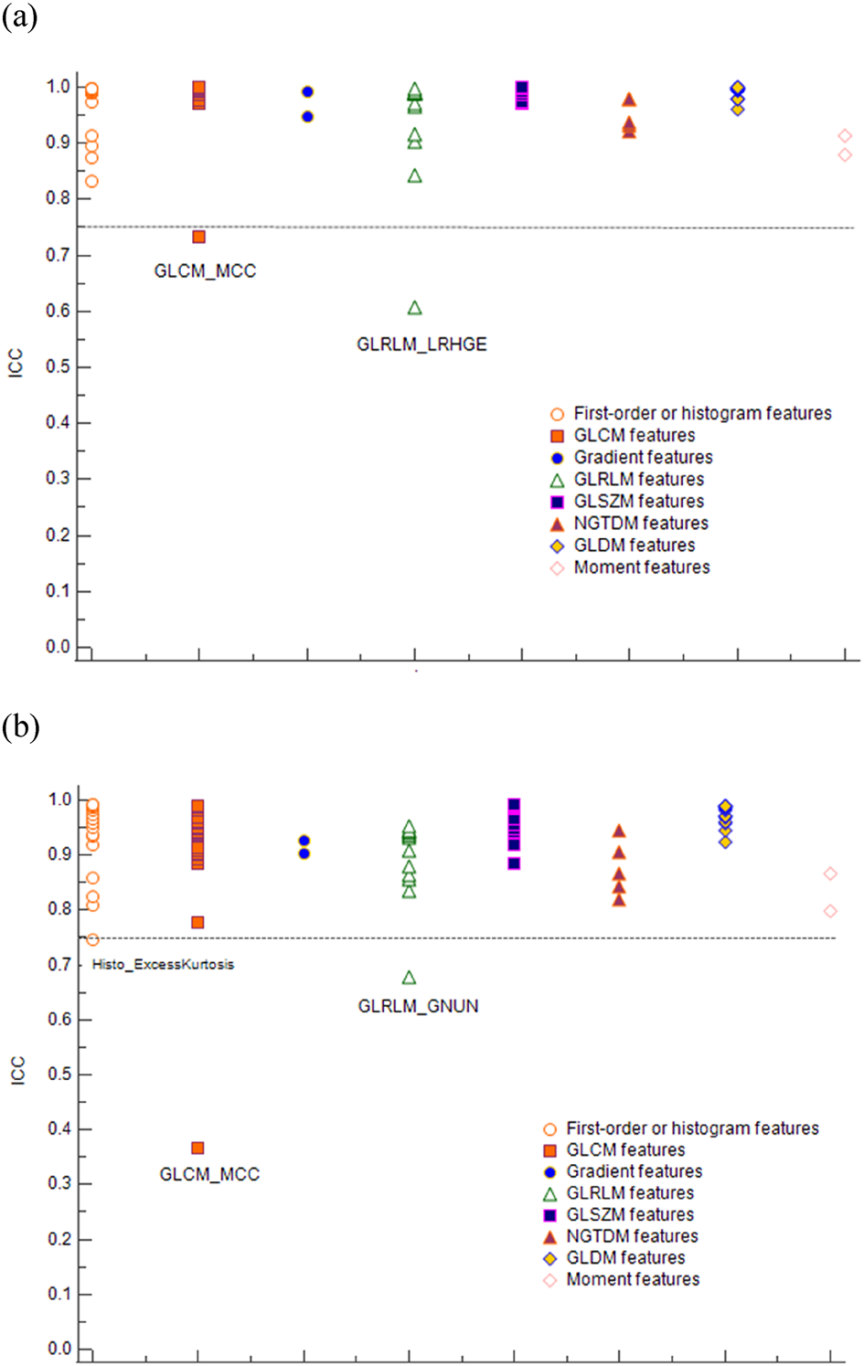
Distribution plot for intraclass correlation of radiomic features of (**a**) myocardium and (**b**) periprosthetic mass. Radiomic features are lined on the x axis and color coded based-on which family of radiomics features they belong to, while their corresponding intraclass correlation coefficient is plotted on the y axis.

Among the 90 radiomic features extracted from LV myocardium, two (GLCM_MCC, GLRLM_LRHGE) showed poor to moderate interobserver agreement (ICC < 0.75) and were excluded from subsequent analysis (Supplemental Table S2 and Fig. 2a). Among the 88 remaining features, 81 features differed significantly between the three reconstruction methods (Supplemental Table S3).

DLR showed significant differences in pairwise comparison with IR in 76 features. The following 12 features did not show significant differences: FirstOrder_RMS, Histo_Mean, Histo_Skewness, Percentile_50, GLCM_InverseVariance, GLCM_Autocor, GLCM_SumAverage, GLRLM_LRLGE, GLDM_HGLE, GLDM_LDLGLE, Moment_J1, and Moment_J2. DLR showed significant differences in pairwise comparison with FBP in 80 features. The following eight features did not show significant differences: Histo_Mean, Percentile_50, GLCM_SumAverage, GLRLM_LRLGE, NGTDM_Strength, GLDM_LDLGLE, Moment_J1, and Moment_J2. IR also showed significant differences in 78 features in a pairwise comparison with FBP. The following ten features did not show significant differences: Histo_Mean, Histo_skewness, Percentile_50, GLCM_CP, GLCM_CS, GLCM_SumAverage, GLRLM_LRLGE, GLDM_LGLE, Moment_J1, and Moment_J2). Seventy-three radiomic features showed significant differences in all three pairwise comparisons.

### Diagnostic values of radiomic features for discrimination of periprosthetic masses

**Figure 3:**
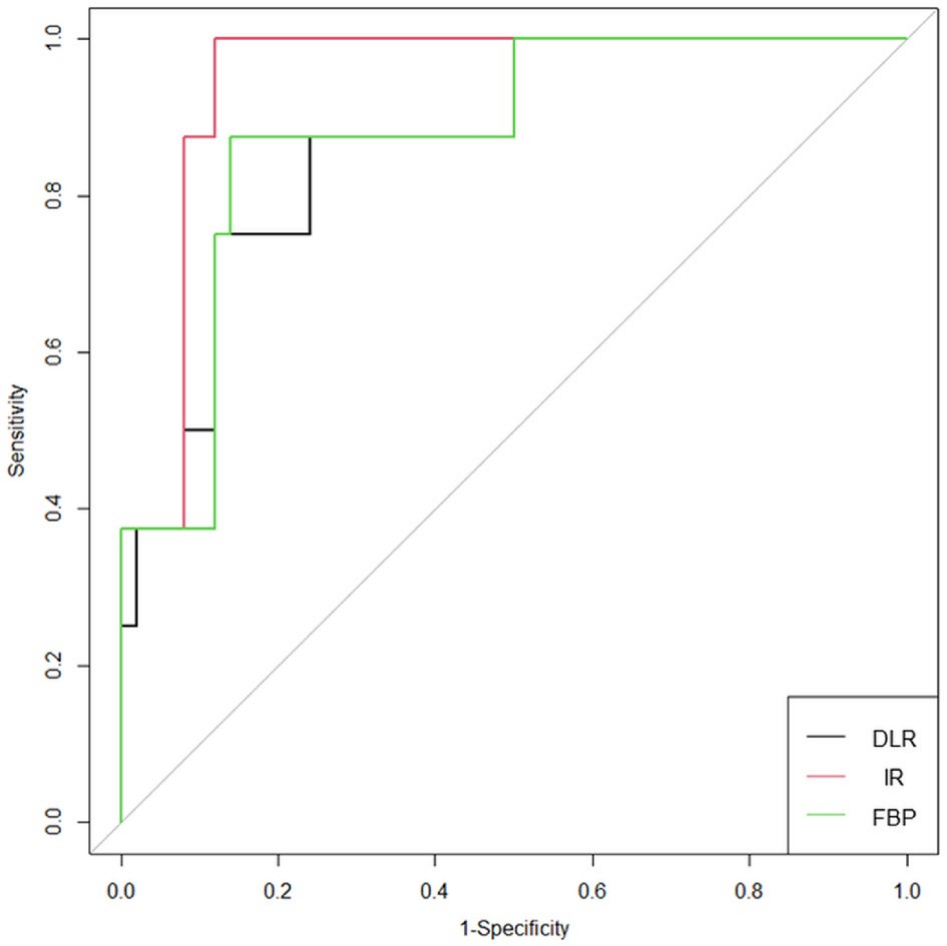
Receiver operating characteristic curves of radiomics models from three different reconstruction methods. DLR, learning-based reconstruction; IR, iterative reconstruction; FBP, filtered back projection.

Three radiomic features (Histo_ExcessKurtosis, GLCM_MCC, and GLRLM_GNUN) exhibited poor to moderate interobserver agreement (ICC < 0.75) and were excluded from subsequent analysis (Fig. 2b). Selected radiomic features were distinct, depending on the reconstruction method (Table 2). The radiomics model based on IR had the best diagnostic performance (AUC, 0.948; 95% CI 0.880–1) relative to DLR (AUC, 0.873; 95% CI 0.735–1) and FBP (AUC, 0.875; 95% CI 0.731–1; Fig. 3), but these differences did not reach significance (*P* > 0.05 for AUC difference; Table 3). Each radiomics model was well calibrated (*P* > 0.999) based on the Hosmer–Lemeshow calibration for goodness-of-fit. When rad-scores from one reconstruction method were validated against another reconstruction method, diagnostic performance differed. For example, rad-score from DLR had an AUC of 0.795 (95% CI 0.573–1) on IR, 0.863 (95% CI 0.703–1) on FBP, and 0.873 (95% CI 0.735–1) on DLR.

**Table 2:** Specifications of the radiomics scores obtained by and radiomics model performance for each reconstruction method.

Model	DLR	IR	FBP
Number of selected features	4	8	6
Name of selected features	GLCM_DiffVariance, GLSZM_LAHGLE, NGTDM_Coarseness, GLDM_SDHGLE	Grad_Mean, Grad_std, GLCM_DiffVariance, GLCM_IMC2, GLSZM_LAHGLE, NGTDM_Coarseness, NGTDM_Busyness, GLDM_DV, GLDM_SDLGLE	Histo_Min,GLCM_HomogeneityNormalized, GLCM_DiffEntropy,GLSZM_ZV, Coarseness, NGTDM_Busyness
Formula for calculation of rad-score	0.058 × GLCM_DiffVariance − 0.015 × GLSZM_LAHGLE + 10.193 × NGTDM_Coarseness + 0.002 × GLDM_SDHGLE	0.012 × Grad_Mean + 0.002 × Grad_std + 0.071 × GLCM_DiffVariance + 0.264 × GLCM_IMC2 − 0.001 × GLSZM_LAHGLE + 139.262 × NGTDM_Coarseness − 3.72 × NGTDM_Busyness − 0.07 × GLDM_DV − 32205.9 × GLDM_SDLGLE	0.002 × Histo_Min − 23.303 × GLCM_HomogeneityNormalized + 3.259 × GLCM_DiffEntropy − 0.282 × GLSZM_ZV + 48.924 × NGTDM_Coarseness − 2.589 × NGTDM_Busyness
AUC (95% CI)	0.873 (0.735, 1)	0.948 (0.880, 1)	0.875 (0.731, 1)
Comparisons of AUC	DLR vs. IR	DLR vs. FBP	IR vs. FBP
AUC difference (95% CI), *P* value	0.075 (− 0.007, 0.157), 0.074	0.002 (− 0.052, 0.057), 0.928	0.073 (− 0.017, 0.162), 0.113

Rad-score, radiomics score; DLR, deep learning-based reconstruction; IR, iterative reconstruction; FBP, filtered back projection; AUC, area under the receiver operating characteristic curve; CI, confidence interval.

**Table 3:** Validating radiomics scores calculated with one reconstruction method in other reconstruction methods.

Reconstruction method for rad-score calculation	DLR			IR			FBP		
Reconstruction method for rad-score validation	DLR	IR	FBP	DLR	IR	FBP	DLR	IR	FBP
AUC (95% CI)	0.873 (0.735, 1)	0.795 (0.573, 1)	0.863 (0.703, 1)	0.902 (0.813, 0.992)	0.948 (0.880, 1)	0.887 (0.730, 1)	0.873 (0.742, 1)	0.853 (0.692, 1)	0.875 (0.731, 1)
AUC difference (95% CI)	N/A	0.078 (− 0.014, 0.169) *P* = 0.096	0.01 (− 0.058, 0.078) *P* = 0.773	0.045 (− 0.001, 0.091) *P* = 0.056	N/A	0.06 (− 0.069, 0.189) *P* = 0.363	0.02 (− 0.059, 0.064) *P* = 0.937	0.022 (− 0.063, 0.108) *P* = 0.608	N/A

Rad-score, radiomics score; DLR, deep learning-based reconstruction; IR, iterative reconstruction; FBP, filtered back projection; AUC, area under the receiver operating characteristic curve; CI, confidence interval; N/A, not-applicable.

## Discussion

Our study demonstrates that CT image quality is better in DLR than IR or FBP, whereas 73 of 88 (83.0%) radiomic features of LV myocardium differ in pairwise comparisons between DLR, IR, and FBP. Each radiomics model exhibits varying performance levels for diagnosing periprosthetic masses, but the differences between models are not statistically significant.

Recent advances in artificial intelligence have led to the development of DLR for CT and its application to various body parts. Most previous studies investigated whether DLR would facilitate noise reduction without altering image quality or diagnostic confidence^18–21,24^. Multiple studies reported that applying DLR to cardiac CT scans reduced noise and improved SNR and CNR more than conventional reconstruction^18,25,26^. Our study shows that DLR produces superior image quality (noise, SNR, and CNR) relative to IR and FBP and is consistent with previous studies.

The reproducibility and model performance of radiomic features are affected by reconstruction methods, and the effects of IR on radiomic features have been investigated^14,15,27,28^. The IR algorithm tended to produce CT images with a more “plastic-looking” texture than FBP reconstructions, so the effects of IR on quantitative radiomic features make sense^29^. Although DLR aims to produce a more natural texture similar to FBP reconstruction, few studies to date have investigated the effects of DLR on quantitative radiomic features^30^. Our study showed that most radiomic features obtained from LV myocardium differ depending on the reconstruction methods and are presumably affected by the reconstruction method. This result is in line with a previous study showing that applying DLR yielded superior feature consistency, discriminative power and repeatability to IR, and FBP for radiomic features on abdominal CT^30^. DLR uses a deep neural network to enhance image quality by removing noise from signal without changing the noise texture itself and is thought to produce adequate images for feature extraction and diagnostic modelling.

In our study, the robustness of radiomics models for diagnosing periprosthetic masses was also affected by the reconstruction method. The selected features used to compute rad-scores varied with each reconstruction method, and the diagnostic performance values (AUC) of each radiomics model differed in pairwise comparisons, even though statistical significance was not reached. The largest AUC in the radiomics model based on IR among the models in our study was an unexpected finding, because DLR showed the best image quality among three kinds of reconstruction methods. However, the better image quality is not always associated with a higher diagnostic performance, as a previous study showed that the use of model-based IR lowers the diagnostic performance for the discrimination of invasive pulmonary adenocarcinomas among subsolid nodules, compared to FBP^31^. To date, the effect of the image reconstruction algorithm on the diagnostic performance of the radiomics model has been scarcely investigated. Moreover, the degree of effects of the reconstruction algorithm on the radiomic features differs according to the anatomic region, strength level of the algorithm, or types of diagnostic task^27,31,32^. Therefore, further comprehensive studies with more anatomic regions or various kinds of diagnostic tasks should be conducted to study the effect of DLR on the diagnostic performance of the radiomics model. Until then, when validating a CT radiomics model for radiologic diagnosis, it is worth remembering that the diagnostic performance is not guaranteed because the reconstruction algorithm has changed.

Our study has several limitations. First, it was conducted in a retrospective manner with a relatively small number of participants, so the generalizability of our results could not be secured. Second, the radiomics models were not validated with external data, and additional studies using data from multiple centers might increase the reliability of the diagnostic performance analysis. Finally, we investigated the effect of only one vendor-specific DLR method, although various approaches have been suggested^17^.

In conclusion, DLR produces myocardial radiomic features that are distinct relative to IR and FBP. Radiomic models based on DLR demonstrate different performances in diagnosing periprosthetic abnormalities than IR or FBP, implying that feature robustness is not guaranteed when applying DLR.

## Methods

### Study population

The Severance Hospital Institutional Review Board approved this study and waived the requirement for informed consent. Our study was conducted in accordance with the Declaration of Helsinki. Our study retrospectively included 387 cardiac CT scans performed with a specific type of CT scanner (Revolution™ CT, GE Healthcare) in 379 consecutive patients between February 2020 and June 2020 at our institution, regardless of indication of CT. Cardiac CT data obtained with this scanner were routinely reconstructed using three methods: DLR, IR, and FBP. CT scans were excluded when reconstructions from any method were not available (n = 102). Finally, 285 CT scans from 284 patients were included in the analysis (Fig. 1).

### CT image acquisition

All cardiac CT examinations were performed using a CT scanner with a 256-slice, 16-cm wide detector (Revolution™ CT, GE Healthcare). The parameters used for scanning were as follows: a prospective electrocardiogram (ECG)-triggering axial mode; tube voltage from attenuation-based tube potential selection software (kV Assist, GE Healthcare), with a baseline of 100 kV; tube current from automated exposure control software (Smart mA, GE Healthcare); and tube rotation time of 280 ms. Beta-blockers to control heart rate were not used. Contrast media was administered using a triple-phase method (5 mL/s injections of 70 mL iopamidol, 30 mL 50% iopamidol in saline, and 20 mL saline). CT angiography was performed with prospective ECG-gating with a padding range of 20%–120% in the R-R interval and acquired 6 s after obtaining 150 HU on the ascending aorta. CT datasets obtained from the best cardiac phases with the least motion artifacts were reconstructed in the axial plane with a slice thickness/interval of 0.625 mm/0.625 mm, using three reconstruction methods for each scan: DLR, “TrueFidelity at high levels” (TF-H); IR, adaptive statistical iterative reconstruction-V (ASIR-V) 60%; and FBP). DLR utilizes a deep neural network-based model to differentiate noise from anatomical structures and emulate high-quality FBP images^33^. In the training process, the DLR engine generates output images from a low-dose input sinogram, compares them with high-dose FBP images from the same objects, and repeatedly fine-tunes the parameters of the deep neural network to suppress image noise, retain the preferred noise texture, and improve spatial resolution.

### CT image analysis

All CT images in each reconstruction group were assessed by two observers (S.H.C., a third-year senior radiology resident, and A.Y.K., a fourth-year medical student) for image quality, image noise, signal-to-noise ratio (SNR), and contrast-to-noise ratio (CNR) by consensus, according to previously described methods^34,35^. Circular regions of interest (ROI) were placed on axial CT images in the ascending aorta immediately cranial to the left coronary ostium by choosing the largest size, excluding the aortic wall. Image noise was defined as the standard deviation of the CT attenuation within the ROI. Four additional circular ROI were placed in the lumen and the adjacent perivascular fat tissue of the left main and the right proximal coronary artery, respectively. SNR and CNR were calculated as follows: $SNR=(\frac{{HU}_{LM}+{HU}_{RCA}}{2})/Noise$;$CNR=\frac{\left[\frac{{HU}_{LM}+{HU}_{RCA}}{2}-\frac{{HU}_{LMPVF}+{HU}_{RCAPVF}}{2}\right]}{Noise},$ where LM is the left main coronary artery; RCA is the proximal right coronary artery, and PVF is perivascular fat.

ROI were independently drawn by two radiologists (Y.J.S., a board-certified radiologist with 12 years of experience in cardiac imaging, and S.H.C., a third-year radiology resident) to segment the left ventricular (LV) myocardium and periprosthetic masses for radiomics analysis. DLR images were segmented using commercially available segmentation software (AVIEW Research, Coreline Soft), and ROIs were copied to IR and FBP reconstructed images.

**Figure 4:**
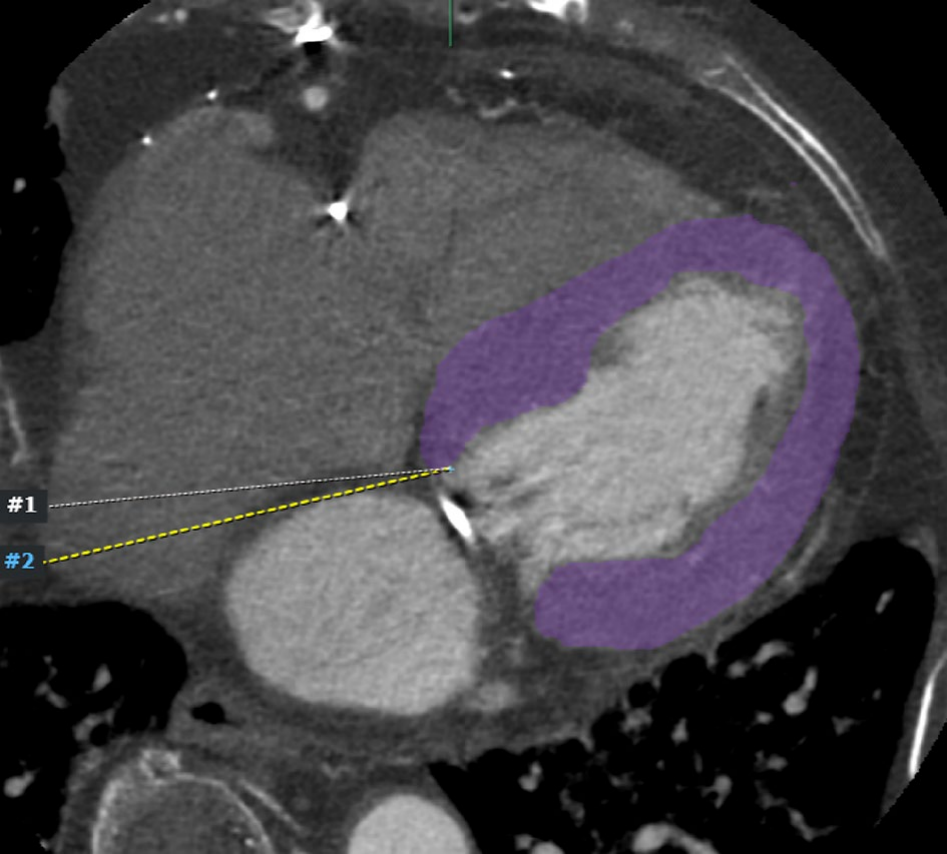
Axial cardiac CT image of a 77-year-old female patient. The myocardium (purple color) is segmented by excluding the LV blood pool and trabeculae to improve reproducibility for delineating the endocardial border. CT, computed tomography; LV, left ventricular.

LV myocardium was segmented by selecting a single slice of an axial CT image at the mid-ventricular myocardial level and drawing an ROI along the LV myocardium, excluding the LV blood pool^15^. Ill-defined blood pools and trabeculae were excluded as much as possible to minimize uncertainty in delineating the endocardium (Fig. 4).

**Figure 5:**
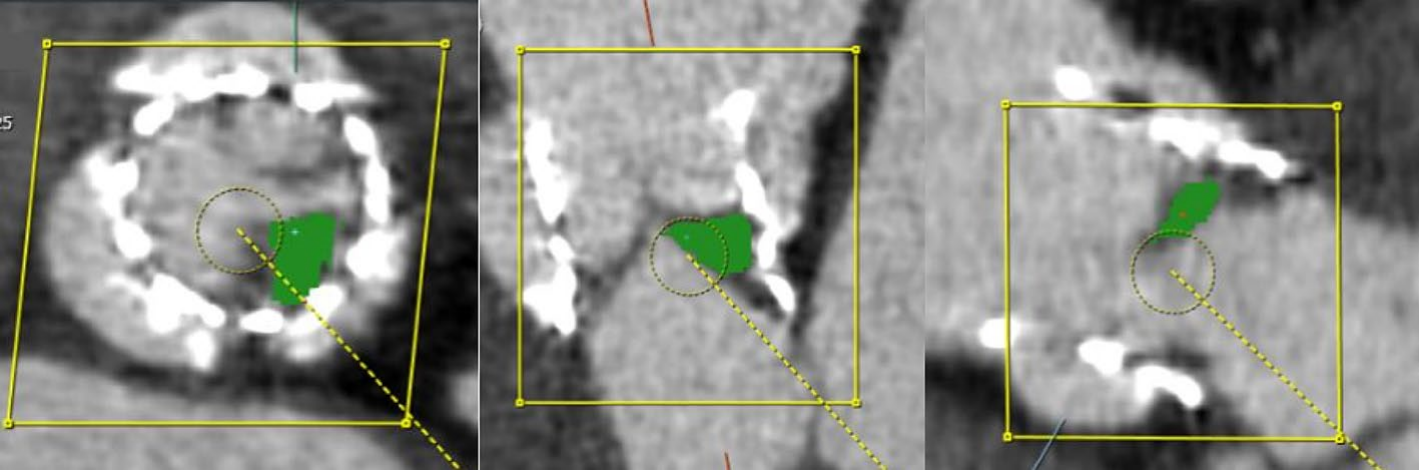
Cardiac CT images of an 83-year-old female exhibiting leaflet thrombosis of the bioprosthetic aortic valve. An ROI is drawn along the hypoattenuated leaflet thickening of the bioprosthetic aortic valve (green color). CT, computed tomography.

Valvular or perivalvular (periprosthetic) masses in patients who had cardiac valve replacement were segmented by reformatting CT images along the short- and long-axes of the prosthetic valve with AVIEW software. Three-dimensional ROI were drawn for periprosthetic masses in images reformatted along the valves’ short-axes. A discrete ROI was drawn for each lesion if a periprosthetic region contained more than one mass (Fig. 5). Calcifications, metal artifacts, and adjacent structures were avoided as much as possible.

### Radiomic feature extraction

A total of 90 radiomic features were extracted from ROI in CT images of LV myocardium and periprosthetic masses: 17 first-order or histogram features, 2 gradient features, 22 Gy-level co-occurrence matrix (GLCM) features, 14 Gy-level run-length matrix (GLRLM) features, 14 Gy-level size zone matrix (GLSZM) features, 5 neighborhood gray-tone difference matrix (NGTDM) features, 14 Gy-level difference matrix (GLDM) features, and 2 moment features. Shape features were not included in the analysis because segmented ROI were identical between reconstruction methods. Feature extraction was performed using AVIEW software, based on the open source program for radiomic analysis, Pyradiomics (Pyradiomics library, version 2.2.0; Computational Imaging and Bioinformatics Lab, Harvard Medical School)^36^. Bin number for intensity discretization was fixed as 64. Intraclass correlation coefficients (ICC) were calculated to evaluate interobserver reproducibility. Features with poor to moderate interobserver reproducibility (ICC < 0.75) were excluded from subsequent analysis^37^.

### Diagnostic value of radiomic features for discriminating periprosthetic masses

Patients were included in a subgroup analysis to evaluate the diagnostic value of radiomic features obtained from the three reconstruction methods if they (a) had undergone cardiac valve surgery with a prosthetic valve (either bioprosthetic or mechanical), (b) exhibited at least one periprosthetic mass in CT images, and (c) the periprosthetic mass(es) were classified as normal, degeneration, thrombus, or pannus during redo cardiac operation or follow-up imaging as a reference standard. For each reconstruction method, radiomics score (rad-score)-based model was constructed to discriminate between abnormal (degeneration, thrombus, or pannus) and normal postoperative changes.

### Statistical analysis

Statistical analyses were performed using R (version 4.0.4.; R Foundation for Statistical Computing, Vienna, Austria) with the “nlme,” “glmnet,” “ResourceSelection,” “rms,” “doBy,” “pheatmap,” and “emmeans” packages. Normally distributed data were identified using the Shapiro–Wilk W test. Image quality parameters (image noise, SNR, and CNR) and the myocardial radiomic features extracted with each reconstruction method were compared using a fitting linear mixed-effects model (LMM) with the reconstruction method as the fixed effect and patients as random effects. False discovery rate control was applied to handle type I error inflation by comparing multiple radiomic features using the Benjamini–Hochberg procedure^38^. Post-hoc *P* value correction for pairwise comparison was done with the Tukey method. Adjusted *P* values under 0.05 were considered significant.

For comparison for discriminating ability of radiomic features for periprosthetic mass, radiomic features were selected by using the least absolute shrinkage and selection operator (LASSO) with tenfold cross-validation for each reconstruction method. A rad-score was calculated using a linear combination of the selected features weighted by each coefficient from the LASSO. To evaluate the performance of the radiomics model (rad-score), generalized linear mixed-effect model (GLMM) was employed to account for the multiple observations per patient. The performance was evaluated using the area under the receiver characteristics (ROC) curve (AUC), and the goodness-of-fit was assessed using Hosmer–Lemeshow calibration. AUCs from the reconstruction methods were compared with the Obuchowski method for clustered data because radiomic features from both observers were used for modeling^39^. The performance of rad-score from one reconstruction method was validated against the other two reconstruction methods.

## Supplementary Information

Supplementary Information.

## Data Availability

The datasets generated during and/or analysed during the current study are available from the corresponding author on reasonable request.

## Abbreviations

CNR: Contrast-to-noise ratio
DLR: Deep learning-based reconstruction
FBP: Filtered back projection
GLMM: Generalized linear mixed-effect model
ICC: Intraclass correlation coefficient
IR: Iterative reconstruction
LASSO: Least absolute shrinkage and selection operator
LV: Left ventricular
SNR: Signal-to-noise ratio
