# Supplementary material for: Deep learning-based reconstruction on cardiac CT yields distinct radiomic features compared to iterative and filtered back projection reconstructions
Source: Sci Rep. 2022 Sep 7;12:15171. doi: 10.1038/s41598-022-19546-1 (PMC9452656; doi:10.1038/s41598-022-19546-1)
Supplement: Supplementary file 1 — Supplementary Information. [file 41598_2022_19546_MOESM1_ESM.docx]

**Supplementary Materials**

**Supplementary Tables**

Table 1. Characteristics of patients who underwent cardiac valve replacement

| Sex | Age (years) | Number of ROI | Final diagnosis |
| --- | --- | --- | --- |
| Female | 77 | 1 | degeneration |
| Male | 74 | 1 | pannus |
| Male | 73 | 1 | degeneration |
| Male | 74 | 1 | degeneration |
| Female | 77 | 1 | pannus |
| Female | 83 | 2 | thrombus |
| Male | 72 | 2 | thrombus |
| Female | 77 | 1 | thrombus |
| Female | 76 | 1 | normal |
| Male | 84 | 1 | thrombus |
| Male | 74 | 2 | thrombus |
| Female | 67 | 3 | normal |
| Female | 80 | 1 | pannus |
| Male | 81 | 1 | thrombus |
| Male | 73 | 3 | thrombus |
| Male | 72 | 2 | thrombus |
| Male | 75 | 2 | thrombus |
| Female | 76 | 1 | pannus |
| Male | 72 | 2 | thrombus |

Table 2. Intraclass correlation coefficients of radiomics features

|  | **LV Myocardium** | **Periprosthetic mass** |
| --- | --- | --- |
| FirstOrder_MAD | 0.994456 | 0.938414 |
| FirstOrder_rMAD | 0.998095 | 0.857905 |
| FirstOrder_RMS | 0.992429 | 0.982183 |
| Histo_Mean | 0.994273 | 0.988028 |
| Histo_Std | 0.990078 | 0.934157 |
| Histo_Skewness | 0.83213 | 0.825617 |
| Histo_ExcessKurtosis | 0.875449 | 0.745583 |
| Histo_Energy | 0.990407 | 0.988943 |
| Histo_Entropy | 0.98833 | 0.949529 |
| Histo_Min | 0.914189 | 0.918067 |
| Histo_Max | 0.896318 | 0.809298 |
| Percentile_10 | 0.99505 | 0.992168 |
| Percentile_25 | 0.998348 | 0.992315 |
| Percentile_50 | 0.996213 | 0.987953 |
| Percentile_75 | 0.99587 | 0.96726 |
| Percentile_90 | 0.991035 | 0.959118 |
| Percentile_95 | 0.973296 | 0.973892 |
| Grad_Mean | 0.99262 | 0.926429 |
| Grad_Std | 0.948343 | 0.902469 |
| GLCM_ASM | 0.994491 | 0.982821 |
| GLCM_IDM | 0.999256 | 0.96987 |
| GLCM_IDMN | 0.998216 | 0.904062 |
| GLCM_Homogeneity | 0.999242 | 0.968968 |
| GLCM_HomogeneityNormalized | 0.999185 | 0.938061 |
| GLCM_InverseVariance | 0.999141 | 0.957835 |
| GLCM_Contrast | 0.998153 | 0.90174 |
| GLCM_Correlation | 0.972121 | 0.891569 |
| GLCM_Autocor | 0.994055 | 0.988623 |
| GLCM_Entropy | 0.994643 | 0.949607 |
| GLCM_CP | 0.996442 | 0.946344 |
| GLCM_CS | 0.992273 | 0.778676 |
| GLCM_CT | 0.996642 | 0.961836 |
| GLCM_SumEntropy | 0.987924 | 0.941936 |
| GLCM_DiffAverage | 0.999151 | 0.933836 |
| GLCM_DiffEntropy | 0.998039 | 0.939472 |
| GLCM_DiffVariance | 0.994298 | 0.885152 |
| GLCM_IMC1 | 0.988123 | 0.912592 |
| GLCM_IMC2 | 0.9772 | 0.907797 |
| GLCM_MCC | 0.732926 | 0.366947 |
| GLCM_MaxProb | 0.997481 | 0.981928 |
| GLCM_SumAverage | 0.994716 | 0.988063 |
| GLRLM_SRE | 0.989942 | 0.941633 |
| GLRLM_LRE | 0.917236 | 0.855325 |
| GLRLM_LGRE | 0.989821 | 0.945674 |
| GLRLM_HGRE | 0.989209 | 0.932792 |
| GLRLM_SRLGE | 0.996426 | 0.943 |
| GLRLM_SRHGE | 0.989713 | 0.944106 |
| GLRLM_LRLGE | 0.843827 | 0.909108 |
| GLRLM_LRHGE | 0.606796 | 0.83582 |
| GLRLM_GNUN | 0.970754 | 0.678026 |
| GLRLM_RLNUN | 0.967 | 0.878893 |
| GLRLM_RP | 0.99036 | 0.93814 |
| GLRLM_RV | 0.903258 | 0.86459 |
| GLRLM_RE | 0.992813 | 0.952039 |
| GLRLM_GLV | 0.992388 | 0.937831 |
| GLSZM_SAE | 0.9988 | 0.946743 |
| GLSZM_LAE | 0.998627 | 0.98406 |
| GLSZM_GLN | 0.971229 | 0.984704 |
| GLSZM_GLNN | 0.987245 | 0.918431 |
| GLSZM_SZN | 0.974827 | 0.986158 |
| GLSZM_SZNN | 0.99956 | 0.956785 |
| GLSZM_ZP | 0.999253 | 0.966008 |
| GLSZM_GLV | 0.992709 | 0.987754 |
| GLSZM_ZV | 0.999463 | 0.989739 |
| GLSZM_ZE | 0.974711 | 0.885348 |
| GLSZM_LGLZE | 0.9988 | 0.946743 |
| GLSZM_HGLZE | 0.998627 | 0.98406 |
| GLSZM_SALGLE | 0.998807 | 0.943384 |
| GLSZM_LAHGLE | 0.998475 | 0.993421 |
| NGTDM_Coarseness | 0.979884 | 0.86689 |
| NGTDM_Contrast | 0.980121 | 0.904976 |
| NGTDM_Busyness | 0.931041 | 0.94438 |
| NGTDM_Complexity | 0.936031 | 0.819275 |
| NGTDM_Strength | 0.921931 | 0.844016 |
| GLDM_SDE | 0.999353 | 0.957747 |
| GLDM_LDE | 0.999097 | 0.988875 |
| GLDM_GLN | 0.980054 | 0.986956 |
| GLDM_DN | 0.962035 | 0.982572 |
| GLDM_DNN | 0.999707 | 0.971658 |
| GLDM_GLV | 0.996274 | 0.945914 |
| GLDM_DV | 0.998223 | 0.967534 |
| GLDM_DE | 0.980134 | 0.924144 |
| GLDM_LGLE | 0.995353 | 0.986217 |
| GLDM_HGLE | 0.993522 | 0.988296 |
| GLDM_SDLGLE | 0.995975 | 0.960557 |
| GLDM_SDHGLE | 0.998552 | 0.971308 |
| GLDM_LDLGLE | 0.98009 | 0.985594 |
| GLDM_LDHGLE | 0.998004 | 0.989514 |
| Moment_J1 | 0.914138 | 0.865554 |
| Moment_J2 | 0.878606 | 0.799414 |

Table 3. Comparison of radiomics features from LV myocardium among three reconstruction groups

| **Radiomics Feature** | **DLR** | **IR** | **FBP** | **Adjusted P-value^*^** | **DLR vs. IR**^†^ | **DLR vs. FBP**^†^ | **IR vs. FBP**^†^ |
| --- | --- | --- | --- | --- | --- | --- | --- |
| FirstOrder_MAD | 41.613 (38.04, 45.186) | 57.279 (53.706, 60.852) | 81.55 (77.977, 85.123) | **<0.001** | **<0.001** | **<0.001** | **<0.001** |
| FirstOrder_rMAD | 26.423 (23.845, 29.002) | 36.709 (34.131, 39.287) | 55.266 (52.688, 57.844) | **<0.001** | **<0.001** | **<0.001** | **<0.001** |
| FirstOrder_RMS | 1171.264 (1166.481, 1176.046) | 1173.088 (1168.306, 1177.871) | 1176.338 (1171.555, 1181.12) | **<0.001** | 0.182 | **<0.001** | **0.005** |
| Histo_Mean | 145.699 (140.699, 150.699) | 145.017 (140.017, 150.017) | 145.932 (140.932, 150.932) | 0.999 | 0.827 | 0.978 | 0.711 |
| Histo_Std | 57.957 (53.413, 62.501) | 77.531 (72.988, 82.075) | 105.893 (101.349, 110.436) | **<0.001** | **<0.001** | **<0.001** | **<0.001** |
| Histo_Skewness | -0.194 (-0.299, -0.09) | -0.123 (-0.228, -0.018) | -0.051 (-0.156, 0.054) | **0.005** | 0.138 | **<0.001** | 0.132 |
| Histo_ExcessKurtosis | 7.699 (6.807, 8.59) | 4.905 (4.013, 5.797) | 1.742 (0.85, 2.634) | **<0.001** | **<0.001** | **<0.001** | **<0.001** |
| Histo_Energy | 0.006 (0.006, 0.006) | 0.005 (0.005, 0.005) | 0.003 (0.003, 0.003) | **<0.001** | **<0.001** | **<0.001** | **<0.001** |
| Histo_Entropy | 7.677 (7.638, 7.716) | 8.024 (7.984, 8.063) | 8.558 (8.519, 8.597) | **<0.001** | **<0.001** | **<0.001** | **<0.001** |
| Histo_Min | -227.38 (-253.681, -201.079) | -283.863 (-310.164, -257.561) | -347.586 (-373.887, -321.285) | **<0.001** | **<0.001** | **<0.001** | **<0.001** |
| Histo_Max | 528.776 (510.436, 547.116) | 566.172 (547.832, 584.513) | 622.185 (603.844, 640.525) | **<0.001** | **<0.001** | **<0.001** | **<0.001** |
| Percentile_10 | 82.852 (74.187, 91.517) | 56.29 (47.625, 64.956) | 17.218 (8.553, 25.884) | **<0.001** | **<0.001** | **<0.001** | **<0.001** |
| Percentile_25 | 114.41 (108.144, 120.676) | 101.48 (95.215, 107.746) | 80.972 (74.706, 87.238) | **<0.001** | **<0.001** | **<0.001** | **<0.001** |
| Percentile_50 | 145.471 (141.114, 149.827) | 146.244 (141.887, 150.6) | 146.668 (142.311, 151.024) | 0.999 | 0.639 | 0.343 | 0.874 |
| Percentile_75 | 177.347 (171.846, 182.849) | 188.791 (183.29, 194.292) | 212.698 (207.196, 218.199) | **<0.001** | **<0.001** | **<0.001** | **<0.001** |
| Percentile_90 | 209.693 (202.489, 216.897) | 232.343 (225.139, 239.546) | 274.538 (267.334, 281.742) | **<0.001** | **<0.001** | **<0.001** | **<0.001** |
| Percentile_95 | 233.271 (224.811, 241.73) | 264.369 (255.91, 272.829) | 313.2 (304.741, 321.66) | **<0.001** | **<0.001** | **<0.001** | **<0.001** |
| Grad_Mean | 45.578 (44.531, 46.625) | 51.608 (50.562, 52.655) | 80.957 (79.911, 82.004) | **<0.001** | **<0.001** | **<0.001** | **<0.001** |
| Grad_Std | 34.542 (33.138, 35.945) | 36.216 (34.813, 37.619) | 47.448 (46.045, 48.852) | **<0.001** | **0.022** | **<0.001** | **<0.001** |
| GLCM_ASM | 0.061 (0.059, 0.062) | 0.036 (0.034, 0.037) | 0.014 (0.013, 0.015) | **<0.001** | **<0.001** | **<0.001** | **<0.001** |
| GLCM_IDM | 0.642 (0.637, 0.646) | 0.565 (0.561, 0.569) | 0.389 (0.385, 0.393) | **<0.001** | **<0.001** | **<0.001** | **<0.001** |
| GLCM_IDMN | 1 (1, 1) | 0.999 (0.999, 0.999) | 0.998 (0.998, 0.998) | **<0.001** | **<0.001** | **<0.001** | **<0.001** |
| GLCM_Homogeneity | 0.663 (0.659, 0.666) | 0.6 (0.597, 0.604) | 0.461 (0.458, 0.465) | **<0.001** | **<0.001** | **<0.001** | **<0.001** |
| GLCM_HomogeneityNormalized | 0.988 (0.987, 0.988) | 0.984 (0.983, 0.984) | 0.971 (0.971, 0.971) | **<0.001** | **<0.001** | **<0.001** | **<0.001** |
| GLCM_InverseVariance | 0.495 (0.492, 0.497) | 0.491 (0.489, 0.493) | 0.383 (0.381, 0.385) | **<0.001** | 0.082 | **<0.001** | **<0.001** |
| GLCM_Contrast | 1.371 (1.261, 1.48) | 2.221 (2.111, 2.33) | 6.498 (6.389, 6.608) | **<0.001** | **<0.001** | **<0.001** | **<0.001** |
| GLCM_Correlation | 0.767 (0.756, 0.779) | 0.756 (0.745, 0.767) | 0.637 (0.626, 0.648) | **<0.001** | **0.01** | **<0.001** | **<0.001** |
| GLCM_Autocor | 1385.57 (1373.998, 1397.141) | 1389.474 (1377.903, 1401.046) | 1396.077 (1384.506, 1407.648) | **0.002** | 0.29 | **<0.001** | **0.03** |
| GLCM_Entropy | 4.783 (4.728, 4.837) | 5.471 (5.417, 5.525) | 6.78 (6.725, 6.834) | **<0.001** | **<0.001** | **<0.001** | **<0.001** |
| GLCM_CP | 2452.765 (-14208.353, 19113.884) | 32026.843 (15365.724, 48687.962) | 47010.703 (30349.585, 63671.822) | **<0.001** | **<0.001** | **<0.001** | 0.079 |
| GLCM_CS | -9.159 (-201.589, 183.27) | -306.909 (-499.338, -114.479) | -337.615 (-530.044, -145.185) | **0.001** | **0.001** | **<0.001** | 0.929 |
| GLCM_CT | 12.603 (4.559, 20.647) | 31.702 (23.658, 39.746) | 51.252 (43.208, 59.296) | **<0.001** | **<0.001** | **<0.001** | **<0.001** |
| GLCM_SumEntropy | 3.625 (3.582, 3.668) | 3.974 (3.931, 4.017) | 4.471 (4.428, 4.514) | **<0.001** | **<0.001** | **<0.001** | **<0.001** |
| GLCM_DiffAverage | 0.818 (0.798, 0.839) | 1.072 (1.052, 1.092) | 1.953 (1.933, 1.973) | **<0.001** | **<0.001** | **<0.001** | **<0.001** |
| GLCM_DiffEntropy | 1.573 (1.556, 1.589) | 1.828 (1.812, 1.845) | 2.502 (2.486, 2.519) | **<0.001** | **<0.001** | **<0.001** | **<0.001** |
| GLCM_DiffVariance | 0.66 (0.612, 0.707) | 1.001 (0.953, 1.048) | 2.52 (2.473, 2.567) | **<0.001** | **<0.001** | **<0.001** | **<0.001** |
| GLCM_IMC1 | -0.244 (-0.25, -0.237) | -0.218 (-0.225, -0.212) | -0.128 (-0.135, -0.122) | **<0.001** | **<0.001** | **<0.001** | **<0.001** |
| GLCM_IMC2 | 0.836 (0.826, 0.846) | 0.828 (0.818, 0.838) | 0.723 (0.713, 0.732) | **<0.001** | **0.035** | **<0.001** | **<0.001** |
| GLCM_MaxProb | 0.14 (0.137, 0.143) | 0.084 (0.082, 0.087) | 0.034 (0.031, 0.036) | **<0.001** | **<0.001** | **<0.001** | **<0.001** |
| GLCM_SumAverage | 74.34 (74.021, 74.659) | 74.293 (73.974, 74.612) | 74.353 (74.034, 74.672) | 0.999 | 0.797 | 0.983 | 0.693 |
| GLRLM_SRE | 0.001 (-0.001, 0.002) | 0.06 (0.059, 0.061) | 0.039 (0.038, 0.04) | **<0.001** | **<0.001** | **<0.001** | **<0.001** |
| GLRLM_LRE | 0.003 (-0.005, 0.012) | 0.263 (0.254, 0.272) | 0.097 (0.088, 0.106) | **<0.001** | **<0.001** | **<0.001** | **<0.001** |
| GLRLM_LGRE | 0 (0, 0) | 0 (0, 0) | 0 (0, 0) | **<0.001** | **<0.001** | **0.002** | **0.019** |
| GLRLM_HGRE | 1.006 (-1.369, 3.38) | 112.889 (110.514, 115.263) | 64.27 (61.895, 66.645) | **<0.001** | **<0.001** | **<0.001** | **<0.001** |
| GLRLM_SRLGE | 0 (0, 0) | 0 (0, 0) | 0 (0, 0) | **<0.001** | **<0.001** | **<0.001** | **0.021** |
| GLRLM_SRHGE | 0.669 (-0.996, 2.333) | 83.589 (81.924, 85.254) | 54.551 (52.887, 56.216) | **<0.001** | **<0.001** | **<0.001** | **<0.001** |
| GLRLM_LRLGE | 0 (-0.002, 0.002) | 0.002 (0, 0.004) | 0 (-0.002, 0.002) | 0.559 | 0.122 | 0.987 | 0.168 |
| GLRLM_GNUN | 0 (0, 0) | 0.001 (0.001, 0.001) | 0 (0, 0) | **<0.001** | **<0.001** | **<0.001** | **<0.001** |
| GLRLM_RLNUN | 0 (0, 0) | 0.004 (0.004, 0.004) | 0.002 (0.001, 0.002) | **<0.001** | **<0.001** | **<0.001** | **<0.001** |
| GLRLM_RP | 0.001 (-0.001, 0.002) | 0.081 (0.08, 0.083) | 0.046 (0.044, 0.048) | **<0.001** | **<0.001** | **<0.001** | **<0.001** |
| GLRLM_RV | 0.003 (-0.005, 0.011) | 0.23 (0.222, 0.237) | 0.088 (0.08, 0.096) | **<0.001** | **<0.001** | **<0.001** | **<0.001** |
| GLRLM_RE | 0.006 (-0.006, 0.017) | 0.633 (0.621, 0.644) | 0.397 (0.386, 0.409) | **<0.001** | **<0.001** | **<0.001** | **<0.001** |
| GLRLM_GLV | 0.802 (-0.924, 2.527) | 93.788 (92.062, 95.514) | 57.729 (56.003, 59.455) | **<0.001** | **<0.001** | **<0.001** | **<0.001** |
| GLSZM_SAE | 0.385 (0.381, 0.39) | 0.457 (0.452, 0.461) | 0.631 (0.627, 0.636) | **<0.001** | **<0.001** | **<0.001** | **<0.001** |
| GLSZM_LAE | 6.366 (6.306, 6.427) | 5.108 (5.048, 5.169) | 3.193 (3.133, 3.254) | **<0.001** | **<0.001** | **<0.001** | **<0.001** |
| GLSZM_GLN | 954.168 (917.471, 990.864) | 851.76 (815.064, 888.456) | 731.173 (694.477, 767.87) | **<0.001** | **<0.001** | **<0.001** | **<0.001** |
| GLSZM_GLNN | 0.174 (0.171, 0.177) | 0.139 (0.135, 0.142) | 0.094 (0.091, 0.097) | **<0.001** | **<0.001** | **<0.001** | **<0.001** |
| GLSZM_SZN | 1497.545 (1423.306, 1571.785) | 1895.908 (1821.669, 1970.147) | 3240.85 (3166.611, 3315.089) | **<0.001** | **<0.001** | **<0.001** | **<0.001** |
| GLSZM_SZNN | 0.272 (0.269, 0.274) | 0.308 (0.305, 0.31) | 0.418 (0.415, 0.421) | **<0.001** | **<0.001** | **<0.001** | **<0.001** |
| GLSZM_ZP | 0.439 (0.436, 0.442) | 0.491 (0.488, 0.494) | 0.623 (0.619, 0.626) | **<0.001** | **<0.001** | **<0.001** | **<0.001** |
| GLSZM_GLV | 1220.001 (1208.384, 1231.618) | 1235.727 (1224.111, 1247.344) | 1268.108 (1256.491, 1279.725) | **<0.001** | **<0.001** | **<0.001** | **<0.001** |
| GLSZM_ZV | 1.1 (1.091, 1.109) | 0.912 (0.903, 0.921) | 0.567 (0.558, 0.576) | **<0.001** | **<0.001** | **<0.001** | **<0.001** |
| GLSZM_ZE | 4.882 (4.846, 4.919) | 5.045 (5.009, 5.082) | 5.155 (5.118, 5.191) | **<0.001** | **<0.001** | **<0.001** | **<0.001** |
| GLSZM_LGLZE | 0.385 (0.381, 0.39) | 0.457 (0.452, 0.461) | 0.631 (0.627, 0.636) | **<0.001** | **<0.001** | **<0.001** | **<0.001** |
| GLSZM_HGLZE | 6.366 (6.306, 6.427) | 5.108 (5.048, 5.169) | 3.193 (3.133, 3.254) | **<0.001** | **<0.001** | **<0.001** | **<0.001** |
| GLSZM_SALGLE | 0.287 (0.282, 0.291) | 0.361 (0.356, 0.365) | 0.555 (0.551, 0.56) | **<0.001** | **<0.001** | **<0.001** | **<0.001** |
| GLSZM_LAHGLE | 71.944 (70.9, 72.987) | 48.554 (47.51, 49.597) | 20.692 (19.648, 21.735) | **<0.001** | **<0.001** | **<0.001** | **<0.001** |
| NGTDM_Coarseness | 0.001 (0.001, 0.001) | 0.001 (0.001, 0.001) | 0.001 (0.001, 0.001) | **<0.001** | **<0.001** | **<0.001** | **<0.001** |
| NGTDM_Contrast | 0.008 (0.006, 0.009) | 0.013 (0.012, 0.014) | 0.031 (0.03, 0.033) | **<0.001** | **<0.001** | **<0.001** | **<0.001** |
| NGTDM_Busyness | 0.882 (0.832, 0.933) | 0.799 (0.748, 0.849) | 0.838 (0.788, 0.889) | **<0.001** | **<0.001** | **0.002** | **0.005** |
| NGTDM_Complexity | 168.404 (139.016, 197.792) | 265.902 (236.514, 295.29) | 552.08 (522.692, 581.468) | **<0.001** | **<0.001** | **<0.001** | **<0.001** |
| NGTDM_Strength | 0.648 (0.551, 0.745) | 0.827 (0.73, 0.924) | 0.631 (0.535, 0.728) | **<0.001** | **<0.001** | 0.897 | **<0.001** |
| GLDM_SDE | 0.147 (0.142, 0.151) | 0.205 (0.2, 0.209) | 0.394 (0.389, 0.398) | **<0.001** | **<0.001** | **<0.001** | **<0.001** |
| GLDM_LDE | 18.759 (18.532, 18.987) | 13.539 (13.312, 13.767) | 6.798 (6.571, 7.025) | **<0.001** | **<0.001** | **<0.001** | **<0.001** |
| GLDM_GLN | 2430.078 (2341.788, 2518.368) | 1872.152 (1783.862, 1960.442) | 1243.747 (1155.457, 1332.037) | **<0.001** | **<0.001** | **<0.001** | **<0.001** |
| GLDM_DN | 2094.535 (2007.08, 2181.99) | 2406.972 (2319.517, 2494.427) | 3331.641 (3244.186, 3419.096) | **<0.001** | **<0.001** | **<0.001** | **<0.001** |
| GLDM_DNN | 0.167 (0.165, 0.168) | 0.192 (0.19, 0.194) | 0.267 (0.266, 0.269) | **<0.001** | **<0.001** | **<0.001** | **<0.001** |
| GLDM_GLV | 3.858 (1.829, 5.886) | 8.916 (6.887, 10.944) | 14.851 (12.822, 16.879) | **<0.001** | **<0.001** | **<0.001** | **<0.001** |
| GLDM_DV | 2.868 (2.842, 2.895) | 2.279 (2.253, 2.305) | 1.296 (1.269, 1.322) | **<0.001** | **<0.001** | **<0.001** | **<0.001** |
| GLDM_DE | 5.428 (5.39, 5.466) | 5.617 (5.579, 5.655) | 5.694 (5.655, 5.732) | **<0.001** | **<0.001** | **<0.001** | **<0.001** |
| GLDM_LGLE | 0.001 (0.001, 0.001) | 0.001 (0.001, 0.001) | 0.001 (0.001, 0.001) | **0.002** | **0.001** | **0.003** | 0.9 |
| GLDM_HGLE | 1386.399 (1374.786, 1398.012) | 1390.901 (1379.288, 1402.514) | 1399.542 (1387.929, 1411.155) | **<0.001** | 0.193 | **<0.001** | **0.003** |
| GLDM_SDLGLE | 0 (0, 0) | 0 (0, 0) | 0 (0, 0) | **<0.001** | **<0.001** | **<0.001** | **<0.001** |
| GLDM_SDHGLE | 205.991 (198.739, 213.244) | 286.284 (279.031, 293.536) | 552.055 (544.803, 559.308) | **<0.001** | **<0.001** | **<0.001** | **<0.001** |
| GLDM_LDLGLE | 0.014 (0.007, 0.021) | 0.02 (0.013, 0.027) | 0.009 (0.002, 0.016) | 0.186 | 0.264 | 0.497 | **0.02** |
| GLDM_LDHGLE | 25828.621 (25519.357, 26137.885) | 18794.545 (18485.281, 19103.809) | 9490.205 (9180.941, 9799.469) | **<0.001** | **<0.001** | **<0.001** | **<0.001** |
| Moment_J1 | 1280.283 (1218.491, 1342.076) | 1279.984 (1218.192, 1341.777) | 1279.987 (1218.195, 1341.78) | 0.999 | >0.999 | >0.999 | >0.999 |
| Moment_J2 | 0.464 (0.409, 0.518) | 0.465 (0.411, 0.52) | 0.465 (0.411, 0.52) | 0.999 | 0.99 | 0.989 | >0.999 |

Data are estimated mean values with the 95% confidential interval in parentheses.

* P values are adjusted by Benjamini–Hochberg procedure for type I error control

^†^ P-value are adjusted by Tukey method.
